# Varicose Veins and Their Complications.—I

**Published:** 1897-10-09

**Authors:** W. McAdam Eccles

**Affiliations:** Assistant Surgeon, West London Hospital, City of London Truss Society, &c.


### VARICOSE VEINS AND THEIR COMPLICATIONS.—I.

By W. McAdam Eccles, M.S. (Lond.), F.R.C.S. (Eng.), Assistant Surgeon, West London Hospital, City of London Truss Society, &c.

Varicosity of veins is a too well known and too widespread an affection not to have been dealt with almost to superfluity, and yet I venture to discuss it again since the complaint so often leads to such grave consequences.

A varicose condition of the vessels which return the de-oxydised blood may occur in the superficial or in the more deeply-placed veins; but the latter, while sometimes leading to œdema of the distal parts, is uncommon, and not, therefore, of very great moment. It is dilatation of superficial vessels which is so very frequent and gives rise to such untoward results.

Some venous channels may be dilated merely as a result of blockage of other veins, and in this case the enlargement is compensatory at first, and but rarely leads to great inconvenience. Such a condition would be very clearly marked on the abdominal wall when the inferior vena cava becomes obstructed. So also the superficial veins of the lower extremity may be much dilated in cases of plugging of the deep veins following parturition, or occurring in the course of specific fevers, &c. This simple secondary dilatation will disappear if its cause be removed, for no permanent lesion to the vessel wall is occasioned until after a lengthened period of distension. However, it is not my purpose to deal with this variety at the present time, but to review the cases where the dilatation is the outcome partly of pressure upon the intermuscular venous trunks, and partly the effect of gravity. Naturally these causes will be most prejudicial in the lower extremity, and it is here that varicosity is so very frequent.

The superficial veins of the thigh and leg lie in the subcutaneous tissue, and are but little supported. They are, moreover, numerous, and their cross sections, if united, would be of large area. They contain numerous valves, but obviously these have a considerable strain thrown upon them, seeing that they have to sustain a very high column of blood. The fact must also be borne in mind that the *vis a tergo* of the heart's action is at its minimum in the distal parts of the lower extremity. Thus it is that, combined with the abnormal pressure liable to be caused by muscular contraction upon the deep veins, the internal saphenous and other superficial veins tend to become dilated.

A loss of tone of the whole body, a debilitated condition in fact, is a very decided predisposing cause of varicose veins. Such a morbid state of the vein walls is very uncommon before puberty, but increases in frequency up to middle age, after which it is less prone to occur. When the varicosity, however, is once fully established it will remain permanent throughout life. There are two chief varieties of varicose veins met with. The first is where one of the larger vessels, and some of its main tributaries constitute the whole or major part of the varicosed veins; the second, in which there are collections of very greatly dilated venules, which are distinctly visible through the skin by the colour of their contained blood. These groups of minute but distended vessels often give rise to severe discomfort. The two species, however, are not infrequently present together.

When a main vein, as the long saphenous, becomes the site of varicosity, its dilatation may occur in one of three different forms. Firstly, the vein may be uniformly enlarged for some distance along its length; this would be the cylindrical variety. Secondly, a part of the vessel may have all its coats dilated in a fusiform manner; this constituting the fusiform variety. Thirdly, a small portion of the vein may be locally dilated, so as to produce a distinct sac, which is very likely to occur opposite a valve, and often the stretched portion causes a marked bulging of the skin over it; this is the sacculated variety.

It must be remembered again, however, that it is by no means infrequent to have the several forms combined in the same patient. As a result of varicosity most grave pathological changes take place in the walls of the veins themselves, and also in the tissues around. The abnormal tension on the vessel wall sooner or later leads to tissue alteration, and this in its turn to physical abnormalities.

The vein is more or less increased in diameter, and at the same time its length becomes greater, which fact accounts for the tortuosity which is so often present. The walls will be found to be thickened in the majority of instances, the increase in tissue being chiefly in the fibrous elements, though sometimes there is definite deposition of additional muscular fibres. Whether this change is the outcome of a chronic inflammation set up by the pressure of the blood within, or is merely a degeneration of muscular fibres, it is difficult to determine. This thickening of the walls may cause them to remain patent, instead of collapsing when cut across. There is, in fact, a similarity in the form and behaviour of the wall of the varicose vein and that of the popliteal vein, which is well known to be usually very much thicker than other veins of the same size.

At certain spots, especially in the region of the valves, the vein is apt to be much thinned, owing to atrophy of its middle coat.

The elasticity of the vein wall, both when thickened and when dilated, is much impaired, and the vessels are unable to contract normally owing to loss of muscular tissue.

If the vein be opened its valves will be seen to be altogether insufficient, and in some advanced cases they are practically entirely absent, being represented by mere ridges. No doubt the dilatation of the vein is to a great extent a reason for the incompetency of the valves, but they are probably also a good deal atrophied by the pressure of blood upon them. The internal coat of the vessel it has been averred is but little altered, but a microscopic section taken transversely across a vein in the site of well-marked varicosity will show that this wall is undoubtedly thickened, but irregularly so. Occasionally distinct atheroma may be present, and secondary calcification is not unknown. The external coat may be thick and opaque.

Varicose veins may give rise to a great amount of discomfort, as they are generally accompanied by a sense of weight and tension in the limb, especially when gravity is allowed to come into play. The aching so often complained of, and most marked at the end of a prolonged period in the upright position, is the outcome of the distension of the vessels, and thus of pressure upon the tissues around and particularly on those of the skin itself.

